# Water level prediction using soft computing techniques: A case study in the Malwathu Oya, Sri Lanka

**DOI:** 10.1371/journal.pone.0282847

**Published:** 2023-04-26

**Authors:** Namal Rathnayake, Upaka Rathnayake, Tuan Linh Dang, Yukinobu Hoshino

**Affiliations:** 1 School of Systems Engineering, Kochi University of Technology, Kochi, Japan; 2 Department of Civil Engineering and Construction, Faculty of Engineering and Design, Atlantic Technological University, Sligo, Ireland; 3 School of Information and Communications Technology, Hanoi University of Science and Technology, Hanoi, Vietnam; KFUPM: King Fahd University of Petroleum & Minerals, SAUDI ARABIA

## Abstract

Hydrologic models to simulate river flows are computationally costly. In addition to the precipitation and other meteorological time series, catchment characteristics, including soil data, land use, land cover, and roughness, are essential in most hydrologic models. The unavailability of these data series challenged the accuracy of simulations. However, recent advances in soft computing techniques offer better approaches and solutions at less computational complexity. These require a minimum amount of data, while they reach higher accuracies depending on the quality of data sets. The Gradient Boosting Algorithms and Adaptive Network-based Fuzzy Inference System (ANFIS) are two such systems that can be used in simulating river flows based on the catchment rainfall. In this paper, the computational capabilities of these two systems were tested in simulated river flows by developing the prediction models for Malwathu Oya in Sri Lanka. The simulated flows were then compared with the ground-measured river flows for accuracy. Correlation of coefficient (R), Per cent-Bias (bias), Nash Sutcliffe Model efficiency (NSE), Mean Absolute Relative Error (MARE), Kling-Gupta Efficiency (KGE), and Root mean square error (RMSE) were used as the comparative indices between Gradient Boosting Algorithms and Adaptive Network-based Fuzzy Inference Systems. Results of the study showcased that both systems can simulate river flows as a function of catchment rainfalls; however, the Cat gradient Boosting algorithm (CatBoost) has a computational edge over the Adaptive Network Based Fuzzy Inference System (ANFIS). The CatBoost algorithm outperformed other algorithms used in this study, with the best correlation score for the testing dataset having 0.9934. The extreme gradient boosting (XGBoost), Light gradient boosting (LightGBM), and Ensemble models scored 0.9283, 0.9253, and 0.9109, respectively. However, more applications should be investigated for sound conclusions.

## Introduction

Globally, floods are the most frequent natural disaster, while their impacts vary from a few households to entire regions [1, 2]. In Sri Lanka, floods are the most frequent natural hazard, similar to the situation worldwide. 28% of the 31,063 catastrophes registered in the nation since 1974 include a flood component. Floods account for 55% of all disaster-related home damages over the same time frame (www.desinventar.net). Therefore, it is not simply a calamity that the nation experiences frequently; it also devastates the economy and communities it affects.

**Fig 1:**
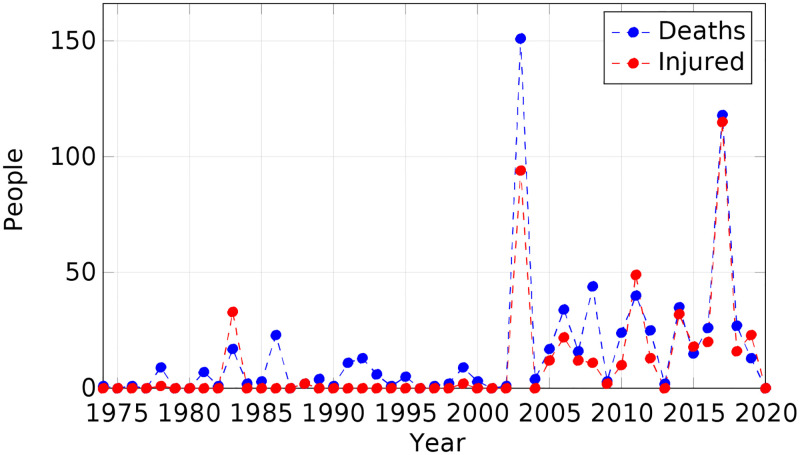
Impact of human lives due to floods during 1974-2020 in Sri Lanka.

Since 1974, 5% of all disaster-related fatalities in the nation have been directly attributable to flooding in the island countries. It is a worrying situation when the Indian Ocean Tsunami of 2004 is taken out of the equation, and 34% of all disaster-related fatalities are attributable to flooding. Floods and other hydro-meteorological disasters are becoming more common in Sri Lanka, according to UNDRR and ADPC [3]. The number of individuals impacted by floods is also increasing, partly due to population expansion, climate-induced rainfall unpredictability, and haphazard development activities that expose more people to flood dangers [4]. Fig 1 shows past flood-related incidents and the destruction caused by them.

Global indices consistently highlight Sri Lanka’s susceptibility to climate change. According to the Global Climate Risk Index, the country will be among the top 10 most vulnerable to climate change in 2018, 2019, and 2020. Regarding nations hit by climate-related disasters worldwide in 2019, the Index placed Sri Lanka second [5]. The country is now more vulnerable to flooding due to climate change-induced severe weather events and climate variability. The nation has 103 significant river basins, and 25 are particularly vulnerable to flooding (Department of Irrigation). Even though most of the nation is contained inside a river basin, rainfall anomalies have led to recurring cycles of both flood and drought, jeopardizing the country’s progress in terms of development. Therefore, many scientific studies have been conducted rapidly during the last few decades to minimize the effect of flooding [6–9].

The World Bank estimates that the country might sustain yearly flood-related losses and damages of up to US$380 million (Global Facility for Disaster Reduction, 2020). To assess the flood-related damages and losses and to determine the needs of the nation for recovery, the Ministries of Disaster Management, National Policies, and Economic Affairs (2016), in partnership with the European Union, UNDP, and the World Bank, conducted thorough post-disaster needs assessments (PDNA). In addition to providing an estimate of the damages, losses, and recovery requirements, these evaluations also identified several weaknesses in Sri Lanka’s disaster risk management system, including the transmission of last-mile early warnings and local disaster response.

The lack of catastrophe risk assessment has significantly impeded the developing of a reliable early warning system, particularly for floods. Despite several attempts, none of the flood risk assessments went beyond just charting the high-risk zones. Such efforts fell short of anticipated outcomes due to a lack of scientifically developed and practically validated methods to evaluate flood hazards and data gaps. Furthermore, the ability to execute large-scale disaster risk reductions, particularly for floods, is constrained by the lack of clarity on the components of disaster risk. Therefore, there is a higher demand for accurate and efficient river flow forecasting method investigation.

Forecasting models use previous data to make accurate predictions regardless of their structural types, such as nonlinear, linear, short, and extended memory. Previous studies’ findings have shown that the results produced by the models such as Autoregressive Integrated Moving Average (ARIMA), Radial Basis Function (RBF) [10], Adaptive Network-based Fuzzy Inference System (ANFIS), and Artificial Neural Network (ANN) were sufficiently accurate and were suitable for forecasting hydrological time series [11–16]. However, in recent studies, gradient boosting-based regression algorithms such as extreme Gradient Boosting (XGBoost) and Light Gradient Boosting (Lightgbm) showed satisfactory results in forecasting problems. Therefore, this study will consider three main river flow forecasting algorithms: Cascaded-ANFIS [17], XGBoost [18], and LightGBM [19].

Moreover, Pandey et al. [20] have researched to predict the seasonal groundwater table depth using Genetic Algorithm optimized Artificial Neural Network (GA-ANN). Their study concluded that using more inputs provides more accurate solutions. Support Vector Machine with radial basis kernel function (SVM-RF) has been used to predict the stage-discharge relationship by Kumar et al. [21]. They state that the SVM-RF performs well and is more robust than the other algorithms used in their study.

For river flow forecasting, researchers have recently used a variety of methods, including radial basis function neural network (RBFNN) and RBFNN-GA [22], Emotional neural network (ENN) [23], multilayer perceptron (MLP), support vector regression (SVR), and random forest (RF) [24], a hybrid approach based on ANN and cooperation search algorithm (2021), convolution neural network (CNN) [25], Hybrid Machine Learning [26], coactive neuro-fuzzy inference system (CANFIS) [25, 27] and finally, a stochastic and neuro-fuzzy-embedded technique [28]. Ebtehaj et al. [29] proposed a novel hybrid method using the Generalized Structure-Group Method of Data Handling (GS-GMDH) and Adaptive Neuro-Fuzzy Inference System with Fuzzy C-Means (ANFIS-FCM) to predict the daily water level. Their study showed that the proposed model is robust for the study region. The absence of attention to nonlinear patterns and factors affecting the flow series is the most obvious flaw in the forecast research that has already been discussed. Regardless of the flow series’ nonlinear structure, they developed models or a robust model for forecasting the flow series.

Additionally, models with comparable structures were considered while evaluating the performance of other models, and the impact of the flow series nonlinear features on models with different structures was not assessed concurrently. This effect is crucial and advantageous because, as several studies have shown [30–32], the flock series’ dynamic properties directly affect the models’ accuracy [33]. Thus, the sensitivities and weaknesses of the models may be identified by considering the flow series’ nonlinear dynamic properties, and the most effective model can be chosen by looking at these aspects. The rationale behind the current study is found here.

According to the comprehensive investigation presented in the above paragraphs, the following flaws of the past studies can be pointed out.

1. The hydrological models developed in past studies highly depend on various complex factors.
2. The machine learning models are mostly based on neural network structure as the base, making the algorithm a black-box learning model. Hence the access to the parameters that control the algorithm is limited (CNN, RBFNN, ARIMA, ANN, and their variances).
3. The black box models are high in computational power and complexity; therefore, using these models in real time is problematic.

Hence, this study focuses on achieving the following objectives.

1. Designing and developing an accurate, low computational complex machine learning model for rainfall-runoff prediction.
2. Conducting comprehensive experiments to support the proposed algorithm using nine regression algorithms (Linear regression, Ridge regression, Lasso regression, Long Short-Term Memory (LSTM), Grated Recurrent Unit (GRU), and Recurrent Neural Networks (RNN), extreme Gradient Boosting (XGBoost), Light Gradient Boosting (LightGBM), Ensemble model).
3. According to the geographical structure of the case study, the usage of the dataset in three different configurations (three lag times such as 1-day, 2-day, and 3-day).
4. Selecting the best rainfall-runoff model based on six hydrological parameters (Correlation Coefficient (R), Percent Bias (basis), Mean Absolute Relative Error (MARE), Kling Gupta Efficiency (KGE), Root Mean Square Error (RMSE), Nash Sutcliffe Model Efficiency (NSE)).

The results of this study provide benchmarks for estimating daily river flow and insight into physical forecasting and the impact of nonlinear time series patterns on model performance.

### Case study: Malwathu Oya—Sri Lanka

**Fig 2:**
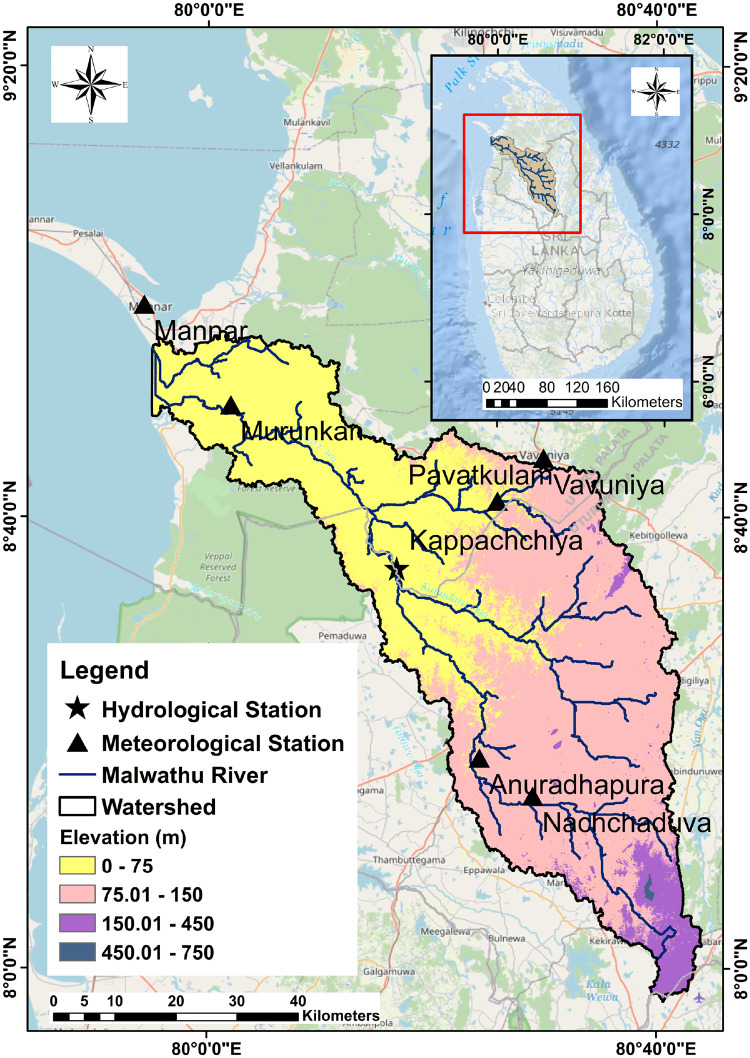
River basin of the Malwathu River in Sri Lanka.

As shown in Fig 2 Malwathu Oya, with a length of 162 km, is the second-largest river in Sri Lanka (catchment area: 3284 km^2^). It rises in the North Central Province’s (766 m MSL) Ritigala Hills and empties into the sea in Arippu in the Mannar District [34]. Districts in Vavuniya and Mannar are traversed by it. A sizeable portion of the basin extends over the Anuradhapura district in the upper catchment before narrowing extensively. There are 1,450 small reservoirs in the basin, while the upper catchment has five big reservoirs. About 410,000 people live in the basin; farmers make up most of them. The poverty headcount index is 7.6, 3.4, and 20.1, respectively, in the districts of Anuradhapura, Vavuniya, and Mannar (Department of Census and Statistics, 2012/13).

In the Malwathu Oya basin, severe flood events have been documented in 2011, 2014, and 2016. The breaching of several small reservoirs caused a significant flood event in December 2014 that is thought to be the worst since 1957, flooding several rural villages in downstream districts of Vavuniya and Mannar [35]. A functional and efficient early warning system is required for the river basin to provide homes at risk of flooding with the time to prepare. The Department of Irrigation is conducting a basin-wide study with money from the World Bank to create a flood model and a hydro-meteorological observation system, hopefully.

## Methodology

This section introduces the overall methodology of the study, including prediction algorithms, the evaluation parameters of the dataset and the models.

### Adaptive Network Based Fuzzy Inference System (ANFIS)

A multi-layer adaptive network-based fuzzy inference system called ANFIS was proposed by Jang [36]. When learning and fine-tuning Fuzzy Inference System (FIS) parameters using a hybrid learning mode, an ANFIS consists of five layers that implement various node functions. The following parameters are updated, and the errors are transferred to the backward pass using the least squared error estimation technique in the forward pass with fixed premise parameters. The backward pass fixes the following parameters while the gradient descent method updates the premise parameters. The assumption and associated parameters for Membership Functions (MF) and FIS will be revealed by repeatedly performing the forward and backward passes. ANIFS is frequently used in various applications such as prediction, forecasting, automatic control, and classification [37]. ANFIS has been extensively used to create novel and hybrid algorithms because of its high performance, and dependability [17, 38, 39]. Additionally, these algorithms have demonstrated promising results in various applications [40–42].

**Fig 3:**
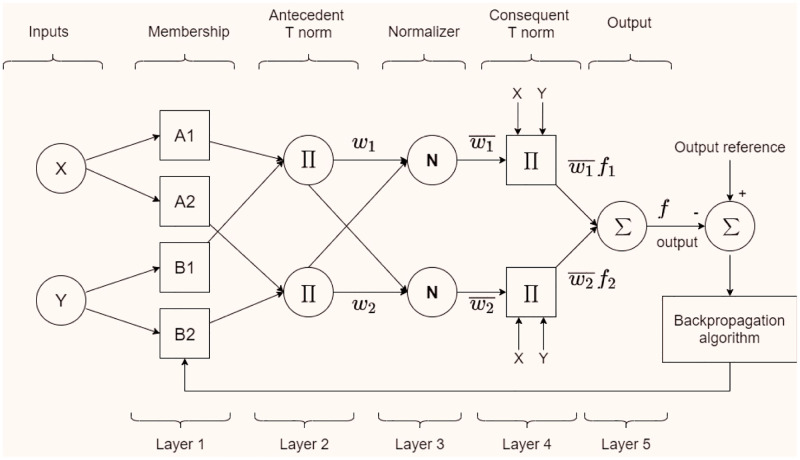
General two-input ANFIS structure.

The general layout of the ANFIS algorithm is depicted in Fig 3. The structure, which has five layers and two inputs and outputs, is depicted in the figure. Layer 1 generates the membership for each input, and the user can predetermine the membership. There are various membership function types, including bell, gaussian, triangular, and trapezoidal. The number of membership functions can vary depending on the problem statement. The figure displays two membership functions—A1, A2, B1 and B2—for each of the inputs X and Y. Before moving on to Layer 3; the following layer creates the Antecedent T-norm for each pair of memberships. Each output produced at the Layer 2 weights (w1 and w2) undergoes normalization. The consequent T-norm is calculated using the initial inputs X and Y. The output is generated at Layer 5 by averaging the outputs from the previous Layers.

### Gradient boosting algorithms

Gradient boosting is a type of machine learning predictive algorithm. It is based on the suspicion that the overall prediction error is minimised when previous models are coupled with the best possible upcoming model. Setting the desired outcomes for this subsequent model is crucial to reducing errors. Each new model advances in the space of potential predictions for each training instance in a manner that reduces prediction error. This technique is called “gradient boosting” because target outcomes are defined for each case based on the gradient of the error about the prediction [43]. Each case’s goal result will differ depending on how changing a case’s forecast affects the overall prediction error.

### Extreme gradient boosting algorithm (XGBoost)

Chen et al. [44] created the XGBoost algorithm. It was developed specifically to increase computational effectiveness and model performance. In an ensemble strategy known as “boosting,” adding more models fixes errors introduced by earlier models. Gradient boosting is a technique that creates new models that predict the residuals of older models combined to produce the final prediction. Gradient-boosting machines are used in a novel and expandable way that has been shown to increase the computational efficiency of boosted tree algorithms. The model addition process is repeated only when there is a noticeable improvement. A gradient descent method reduces the loss when adding new models. In 2015, XGBoost had finished 17 of the 29 machine-learning projects submitted to Kaggle. Speed was significantly boosted by using many CPU cores and reducing the look-up times of individual trees created with XGBoost. This method is constructed in R and Python using the SciKit-Learn [45] package and uses novel regularization techniques.

### Light gradient boosting algorithm (LightGBM)

The LightGBM [46] algorithm from Microsoft is an open-source GBDT. The histogram-based algorithm is the foundation for the parallel voting decision tree technique, which speeds up training, uses less memory, and integrates complex network connectivity to maximize parallel learning [47, 48]. At each iteration, the local voting choice for the top k characteristics and the global voting decision for the top 2k attributes are made. The LightGBM uses the leaf-wise method to determine which leaf has the most significant splitter gain.

### Cat gradient boosting algorithm (CatBoost)

Gradient boosting is a powerful machine-learning method that can handle problems with various features, noisy data, and complex interactions. CatBoost, a machine learning method based on gradient-boosting decision trees (GBDT), was introduced by Yandex developers in 2017 [49].

CatBoost has advantages over other GBDT algorithms: 1. The method effectively handles category features. Traditional GBDT methods can replace categorical traits with fair average label values. 2. CatBoost combines many category properties. CatBoost applies a greedy approach to integrate all categorical traits and combinations in the current tree with all categorical features in the dataset.

CatBoost can be used to address gradient bias. Each iteration of GBDT produces a weak learner, and each learner is taught using the gradient of the preceding learner. The total findings from each learner’s categorization make up the output [50].

**Fig 4:**
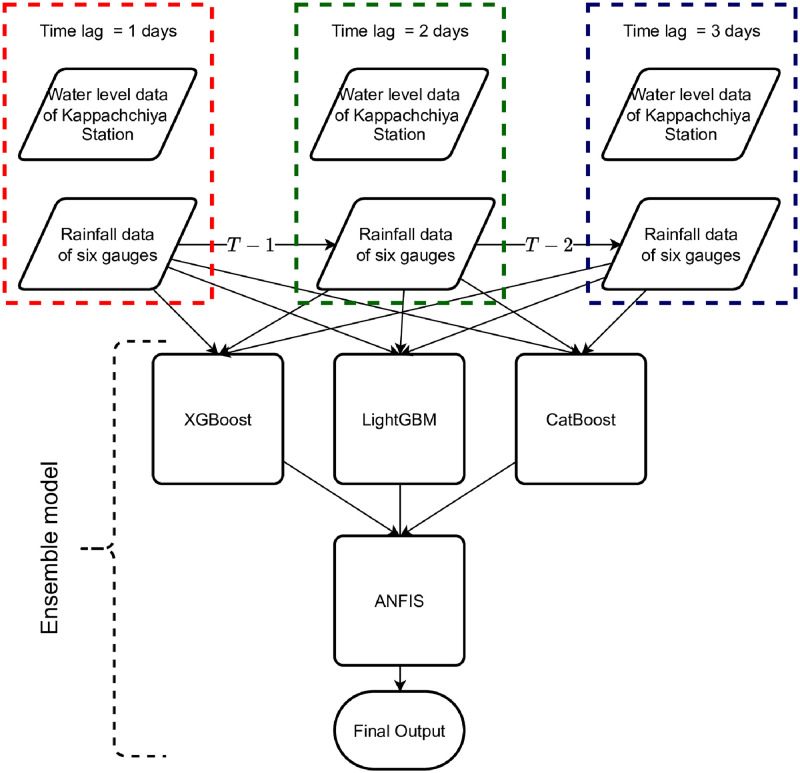
The summarized flow of the methodology.

As shown in Fig 4, the overall flow of the methodology is presented. The inputs are considered to be taken with three different time lags one day, two days, and three days. The experiments were conducted with varying dimensions of input to check the variance of the relationship between the rainfall and water level with the time lags. As in the figure, the final model combines three gradient boosting algorithms: XGBoost, LigthGBM, CatBoost and a final regressor called ANFIS.

### Model parameter tuning

This study’s parameter tuning of the selected algorithm was done according to Table 1. Each of the algorithm parameters was introduced separately concerning the algorithm. The tuning was done by repeating the task multiple times until the best configuration of parameters was achieved.

**Table 1:** Machine learning algorithm parameters.

Algorithm	Parameter	Value
XGBoost	objective	reg:linear
	colsample_bytree	0.3
	learning_rate	0.01
	max_depth	5
	Alpha	10
	n_estimators	10
CatBoost	Iterations	500
	learning_rate	0.01
	eval_metric	MultiClass
	sampling_frequency	PerTree
	penalties_coefficient	1
	max_leaves	64
	permutation_count	4
	Depth	6
LightGBM	num_leaves	31
	objective	binary
	learning_rate	0.01
	boosting_type	Dart
ANFIS	Membership_Function	Bell
	Number_of_MFs	3
	Number_of_Inputs	3
	Iterations	100

### Dataset

The dataset used in this study was a combination of six rainfall gauges as inputs and water level station data as an output. Here, Murunkan, Pavattakulam, Nachchiduva, Vavuniya, Mannar, and Anuradhapura (Apura) rain gauges were considered the inputs (unit in centimetres). The Water level measurement at the Kappachichiya location was taken as the output (unit in meters). The data was collected between 2005 and 2018 and recorded as daily rainfall data and water level data. The total number of samples at each input and output variable was 4765. The dataset was divided into training and testing with 70% to 30% ratios. The overall descriptive analysis is shown in Table 2.

**Table 2:** Descriptive analysis of the dataset of the Malwathu River—Sri Lanka.

	Murunkan	Pavattakulam	Nachchiduva	Vavniya	Mannar	Apura	Kappachichiya
Sample count	4765	4765	4765	4765	4765	4765	4765
MEAN	2.55	3.08	4.18	3.91	2.58	4.09	0.16
STD	9.76	11.24	17.08	12.89	11.15	13.04	0.51
MIN	0	0	0	0	0	0	0.0025
MAX	161.50	185.00	417.97	225.70	350.90	192.50	6.20

**Fig 5:**
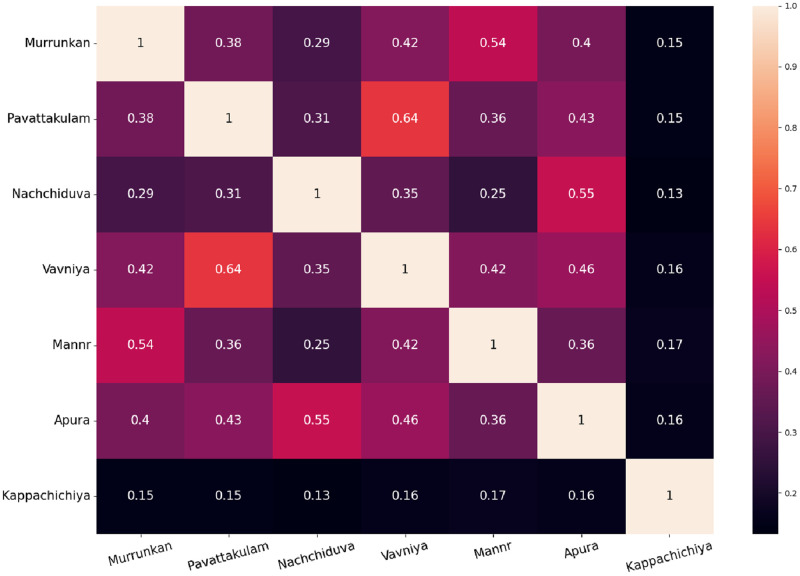
Correlation presentation of the Malwathu river dataset.

Moreover, Fig 5 shows the correlation calculation of the dataset used in this study. Here, the Kappachchiya data point is the water level station variable. It can be seen that the relationship between the water level measurement point and the rainfall measurements is shallow. However, the correlation between the rainfall data shows a significant relationship.

### Statistical evaluation criterion

The evaluation of the results of this study was done by associating the following parameters. The statistical evaluation parameters accomplished the evaluation of the models. The equations below introduce the computations of above mentioned statical evaluation parameters.

1. Correlation of coefficient (R) (Eq 1)
  $$\begin{array}{c} R=\frac{\sum \left(v\left(t\right)-\bar{v}\left(t\right)\right)\left(u\left(t\right)-\bar{u}\left(t\right)\right)}{\sqrt{\sum {\left(v\left(t\right)-\bar{v}\left(t\right)\right)}^{2}\sum {\left(u\left(t\right)-\bar{u}\left(t\right)\right)}^{2}}} \end{array}$$ (1)
2. Percent-Bias (bias) (Eq 2)
  $$\begin{array}{c} bias=\frac{\sum_{j=1}^{k}u\left(t\right)-\bar{u}\left(t\right)}{\sum_{j=1}^{k}u\left(t\right)} \end{array}$$ (2)
3. Nash Sutcliffe model efficiency (NSE) (Eq 3)
  $$\begin{array}{c} NSE=1-\frac{\sum_{j=1}^{k}{\left(u\left(t\right)-\bar{u}\left(t\right)\right)}^{2}}{\sum_{j=1}^{k}{\left(u\left(t\right)-\bar{v}\left(t\right)\right)}^{2}} \end{array}$$ (3)
4. Mean Absolute Relative Error (MARE) (Eq 4)
  $$\begin{array}{c} MARE=\frac{\sum_{j=1}^{k}\left|e_{j}-s_{j}\right|}{\sum_{j=1}^{k}e_{j}} \end{array}$$ (4)
5. Kling-Gupta Efficiency (KGE) (Eq 5)
  $$\begin{array}{c} \begin{array}{c} KGE=1-\sqrt{{\left[r-1\right]}^{2}+{\left[\alpha -1\right]}^{2}+{\left[\beta -1\right]}^{2}}\\ r=\frac{cov(e,s)}{\sigma (e)\cdot \sigma (s)}\\ \alpha =\frac{\sigma (s)}{\sigma (e)}\\ \beta =\frac{\mu (s)}{\mu (e)} \end{array} \end{array}$$ (5)
6. Root Mean Square Error (RMSE) (Eq 6)
  $$\begin{array}{c} RMSE=\sqrt{\frac{1}{q}\sum_{t=1}^{q}{\left(u\left(t\right)-\bar{u}\left(t\right)\right)}^{2}} \end{array}$$ (6)

Here, for Eqs 1–6, *u*(*t*) is the predicted parameter, $\bar{u}(t$) is the mean of predicted parameter *v*(*t*) is the measured parameter, *k* is the population size, and $\bar{v}(t$) is the mean of the measured parameter. The correlation coefficient (R) represents the goodness of fit. It varies from -1 to 1; the best is when it becomes 1. Bias tells the differences between predicted to measured values. It assesses the simulated values’ average propensity to be greater or lower than their observed values. The ideal bias value is 0, and 1 becomes the worst. NSE calculates the perfectness of the match between actual and prediction. The results of the NSE can vary between minus infinity being the worst and 1 being the ideal [51].

## Results and discussion

This section showcases the model predictability of the water level against rainfall. Each algorithm’s performance is discussed separately, and an overall comparative analysis is presented. Here, the outputs of the gradient boosting models were considered the inputs of the ANFIS algorithm due to the curse of dimensionality issue of the ANFIS algorithm. Therefore, it can be stated that the ANFIS model provides an Ensemble structure where the gradient boosting algorithms (XGBoost, CatBoost, and LightGBM) are base algorithms, and the ANFIS is the final estimator.

The experiments were conducted with three different usages of the dataset as follows.

1. With one day before past data (*t* + *t*_−1_)
2. With two days before past data (*t* + *t*_−1_ + *t*_−2_)
3. With three days before past data (*t* + *t*_−1_ + *t*_−2_+ *t*_−3_)

These lag times were decided according to the civil engineering expertise of water flow concerning the geographical structure of the case study.

### Extreme gradient boosting algorithm (XGBoost)

**Fig 6:**
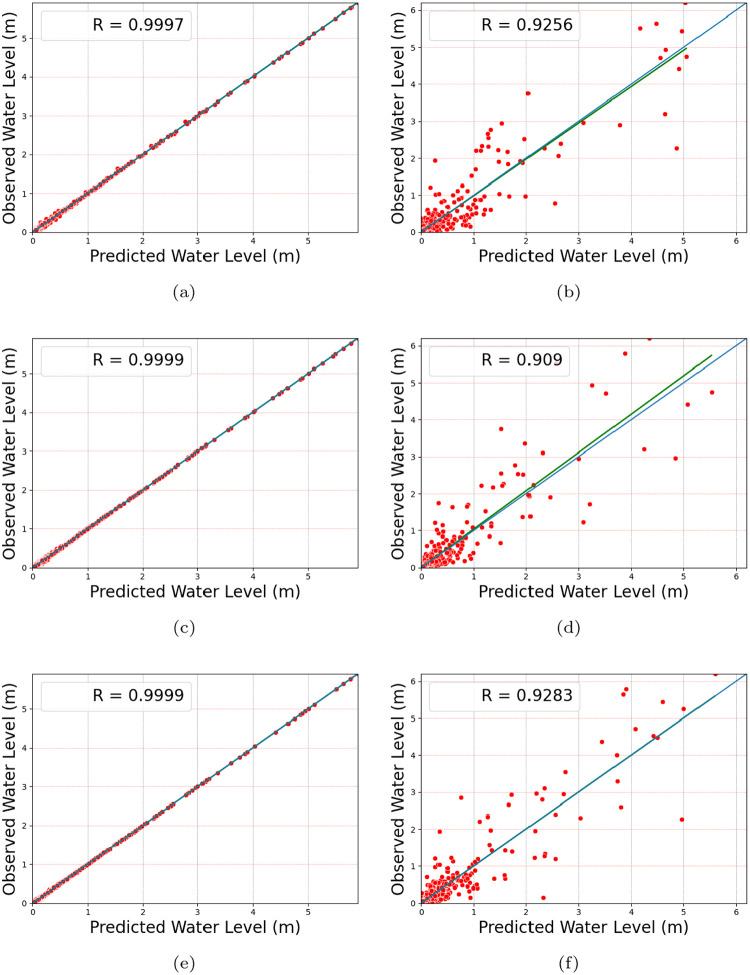
Comparission between predicted and the actual water level for water levels of Malwathu River in Sri Lanka using XGBoost algorithm. (a) With one day before past data (Training); (b) With one day before past data (Testing); (c) With two days before past data (Training); (d) With two days before past data (Testing); (e) With three days before past data (Training); (f) With three days before past data (Testing).

The performances of the XGBoost algorithm are presented in this section. Fig 6 shows that the water level prediction is plotted against the time instances. As stated before, the experiments were conducted at three different lag times. The correlation coefficient (R) at each occurrence is shown in the figure. In the XGBoost model, the highest R score was given by the three days past dataset with 0.9283 for the testing. It is also noticeable that the R Score varies excitingly with the lag time. The highest to lowest R scores were presented as 3 − *day* > 1 − *day* > 2 − *day* configurations.

### Light gradient boosting algorithm (LightGBM)

**Fig 7:**
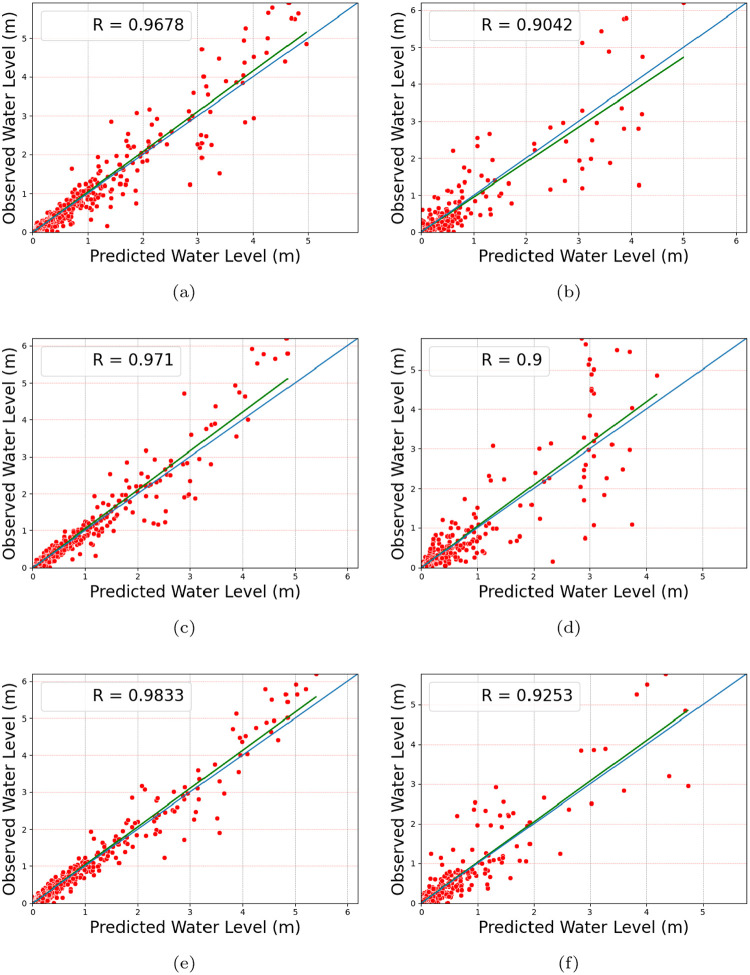
Comparission between predicted and the actual water level for water levels of Malwathu River in Sri Lanka using LightGBM algorithm. (a) With one day before past data (Training); (b) With one day before past data (Testing); (c) With two days before past data (Training); (d) With two days before past data (Testing); (e) With three days before past data (Training); (f) With three days before past data (Testing).

The performances of the LightGBM algorithm are presented in this section. Fig 7 shows that the water level prediction is plotted against the time instances. As stated before, the experiments were conducted at three different lag times. The correlation coefficient (R) at each occurrence is shown in the figure. In the LightGBM model, the highest R score was given by the 3-day past dataset with 0.9253 for the testing. It is also noticeable that the R Score varies excitingly with the lag time. The highest to lowest R scores were presented as 3 − *day* > 1 − *day* > 2 − *day* configurations.

### Cat gradient boosting algorithm (CatBoost)

**Fig 8:**
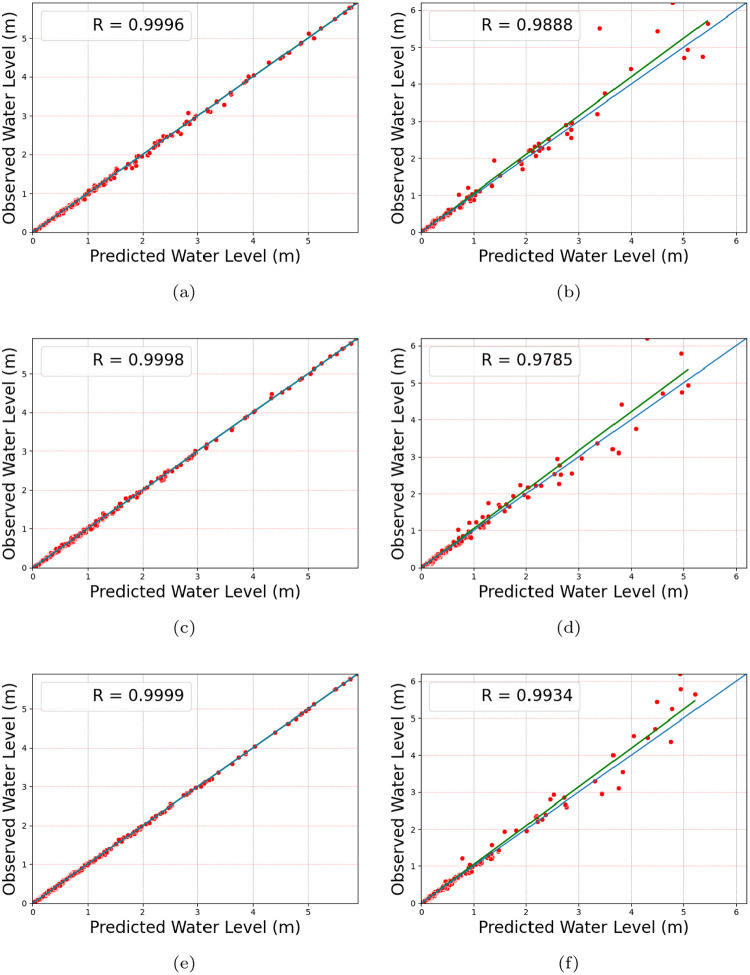
Comparison between predicted and the actual water level for water levels of Malwathu River in Sri Lanka using CatBoost algorithm. (a) With one day before past data (Training); (b) With one day before past data (Testing); (c) With two days before past data (Training); (d) With two days before past data (Testing); (e) With three days before past data (Training); (f) With three days before past data (Testing)).

The performances of the CatBoost algorithm are presented in this section. Fig 8 shows that the water level prediction is plotted against the time instances. As stated before, the experiments were conducted at three different lag times. The correlation coefficient (R) at each occurrence is shown in the figure. In the CatBoost model, the highest R score was given by the 3-day past dataset with 0.9934 for the testing. It is also noticeable that the R Score varies excitingly with the lag time. The highest to lowest R scores were presented as 3 − *day* > 1 − *day* > 2 − *day* configurations, which was the same pattern as XGBoost.

### Adaptive Network Based Fuzzy Inference System (ANFIS) and gradient boosting methods as the ensemble model

**Fig 9:**
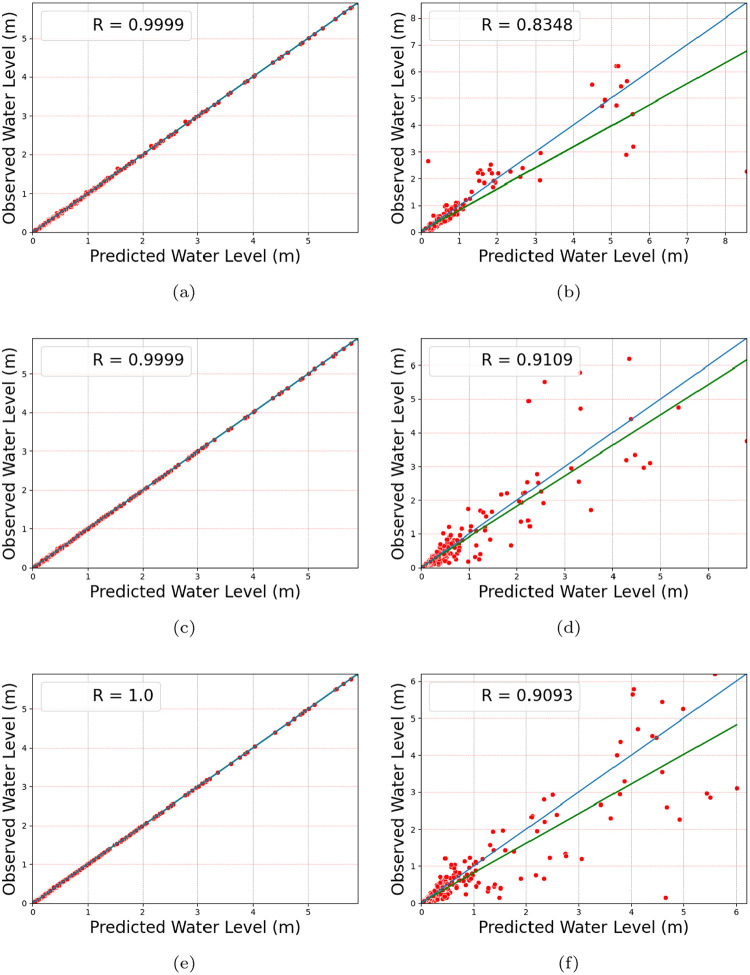
Comparison between predicted and the actual water level for water levels of Malwathu River in Sri Lanka using ensemble algorithm. (a) With one day before past data (Training); (b) With one day before past data (Testing); (c) With two days before past data (Training); (d) With two days before past data (Testing); (e) With three days before past data (Training); (f) With three days before past data (Testing).

The performances of the ensemble algorithm are presented in this section. Fig 9 shows that the water level prediction is plotted against the time instances. As stated before, the experiments were conducted at three different lag times. The correlation coefficient (R) at each occurrence is shown in the figure. In the ensemble model, the highest R score was given by the 2-day past dataset with 0.9109 for the testing. It is also noticeable that the R Score varies excitingly with the lag time. The highest to lowest R scores were presented as 2 − *day* > 3 − *day* > 1 − *day* configurations.

### Comparison with state-of-the-art regression models

Moreover, State-of-the-art algorithms were considered in this study to enhance the comparative analysis of the results. The following algorithms were considered state-of-the-art.

1. Grated Recurrent Unit (GRU)
2. Long Short Time Memory (LSTM)
3. Recurrent Neural Networks (RNN)
4. Lasso Regression (Lasso)
5. Linear Regression (Linear)

Hyper-parameter tuning methods tuned the parameters of the algorithms mentioned above.

Table 3 shows a comprehensive performance evaluation of nine different algorithms. These algorithms were used to develop a model for the Malwathu River water level prediction. As in the Table, three combinations of the dataset were used, and five statical parameters were calculated as Percent Bias (Bias), Mean Absolute Relative Error (MARE), Root Mean Square Error (RMSE), Nash-Sutcliffe Efficiency (NSE), and Kling-Gupta Efficiency (KGE).

**Table 3:** State-of-the-art regression models performances evaluation.

Algorithm	Configuration	Bias	MARE	RMSE	NSE	KGE
CatBoost	1-day	2.64	0.08	0.08	0.98	0.94
	2-day	2.57	0.10	0.10	0.95	0.92
	3-day	1.60	0.08	0.07	0.98	0.95
Ensemble	1-day	5.24	0.27	0.32	0.64	0.82
	2-day	-4.15	0.27	0.21	0.82	0.90
	3-day	-14.17	0.31	0.26	0.77	0.78
GRU	1-day	18.81	0.35	0.20	0.85	0.73
	2-day	6.36	0.30	0.19	0.86	0.84
	3-day	-0.31	0.31	0.19	0.86	0.88
Lasso Regression	1-day	-0.16	0.45	0.20	0.85	0.89
	2-day	-0.12	0.65	0.24	0.77	0.87
	3-day	1.28	0.76	0.26	0.73	0.85
LightGBM	1-day	-0.35	0.47	0.22	0.82	0.59
	2-day	-3.66	0.47	0.20	0.83	0.62
	3-day	0.53	0.45	0.23	0.82	0.62
LSTM	1-day	10.14	0.31	0.19	0.86	0.81
	2-day	14.64	0.32	0.19	0.86	0.77
	3-day	1.99	0.30	0.18	0.87	0.87
Linear Regression	1-day	-0.16	0.42	0.19	0.85	0.89
	2-day	-0.08	0.40	0.19	0.86	0.90
	3-day	0.57	0.41	0.19	0.86	0.88
RNN	1-day	12.35	0.36	0.20	0.84	0.79
	2-day	21.63	0.36	0.21	0.83	0.70
	3-day	19.15	0.39	0.21	0.82	0.73
XGBoost	1-day	1.31	0.35	0.19	0.86	0.91
	2-day	4.29	0.35	0.21	0.82	0.84
	3-day	0.53	0.33	0.20	0.86	0.90

The lowest bias value of the experiments was shown by the Linear Regression algorithm model with -0.08 at the 3-day configuration, while RNN showed the highest bias value of 21.63 at 2-day configuration. However, the gradient-boosting methods showed competitive bias values of 1.60, 0.53, and 0.53 when using CatBoost, LightGBM, and XGBoost algorithms, respectively.

The MARE and RMSE values were similar for all most all the algorithms. CatBoost Algorithm scored the lowest MARE and RMSE with 0.08 and 0.07, respectively. The most significant MARE value is 0.76 for lasso regression at a 3-day configuration. The ensemble model gave the highest RMSE with 0.32 at 1-day configuration. Overall for the error-wise evaluation, the gradient boosting algorithm gives the lowest than the black-box models.

When comparing each algorithm’s NSE and KGE values, the highest value is given by the CatBoost algorithm with 0.98 and 0.95, respectively.

Overall, these results convey that the CatBoost algorithm, a gradient-boosting algorithm, outperforms the other algorithms in almost all evaluation criteria. It is also noticeable that the ensemble model performance does not provide a higher state when compared with the other algorithms.

## Conclusion

Simulating hydrological models is high in computational cost due to the numerous data points and input-output dimensions. Though black-box algorithms perform well in the literature for prediction and forecasting, the excessive use of computational resources is challenging to handle. Therefore, this study was conducted to evaluate and analyze the computational performances of the gradient-boosting algorithms in hydrological prediction and forecasting. CatBoost, extreme boost and light gradient boost algorithms were considered gradient-boosting algorithms due to their vast popularity in the scientific community.

This study focuses on a specific case study called Malwathu River, located in Sri Lanka. There have been vast amounts of human and infrastructure damage due to this river’s sudden flooding. Therefore, this work utilizes the rainfall and water level dataset of the Malwathu River to train hydrological models to predict and forecast the river’s flooding. The data was collected from the year 2005 to 2018 with six rainfall gauges in the river basin of the Malwathu River. Moreover, three configurations were established for the experiments with 1-day, 2-day, and 3-day lag times.

The results were evaluated under six statical evaluation criteria. Namely, correlation of coefficient (R), Per cent-Bias (bias), Nash Sutcliffe Model efficiency coefficient (NSE), Mean Absolute Relative Error (MARE), Kling-Gupta Efficiency (KGE), and Root mean square error (RMSE). These evaluation criteria are well-reputed for hydrological model analysis in the literature. Moreover, an Adaptive network-based fuzzy Inference system (ANFIS) based ensemble model was generated to check if the performance can be enhanced using gradient boosting algorithms as base models.

The results of this study show that the CatBoost hydrological model outperforms other algorithms. The results were compared with nine different algorithms, including black-box algorithms (LSTM, GRU, RNN). This study concludes that the Cat gradient boosting algorithm can predict the hydrological modelling better than the general black-box algorithms. As for the future aspect, implementing a real-time flood warning system in Sri Lanka using a model that consumes less power and computational cost can be introduced. It is also noticeable that the Ensemble model performs comparatively better than LSTM, GRU, RNN, and other algorithms.

One of the main objectives of this study was to implement an Ensemble model using three gradient-boosting algorithms and an ANFIS model. Although the Ensemble model does not provide the best results, there is a chance of this model working well for other case studies. Therefore, as for the limitations of this study, the lack of study cases and applications to check the performances of used algorithms can be introduced. Therefore, implementing a generalized rainfall-runoff predicting model for the South Asia region can be presented for future objectives.
